# Increased Nucleus Accumbens Volume in College Binge Drinkers - Preliminary Evidence From Manually Segmented MRI Analysis

**DOI:** 10.3389/fpsyt.2019.01005

**Published:** 2020-02-10

**Authors:** Sónia S. Sousa, Adriana Sampaio, Eduardo López-Caneda, Clothilde Bec, Óscar F. Gonçalves, Alberto Crego

**Affiliations:** ^1^Psychological Neuroscience Lab, CIPsi, School of Psychology, University of Minho, Braga, Portugal; ^2^Spaulding Neuromodulation Center, Department of Physical Medicine and Rehabilitation, Spaulding Rehabilitation Hospital and Massachusetts General Hospital, Harvard Medical School, Boston, MA, United States; ^3^Department of Applied Psychology, Bouvé College of Health Sciences, Northeastern University, Boston, MA, United States

**Keywords:** alcohol, binge drinking, college students, nucleus accumbens, nucleus caudate, striatum, MRI manual segmentation

## Abstract

**Introduction:**

Binge drinking (BD) is characterized by high alcohol intake in a short time followed by periods of withdrawal. This pattern is very common during adolescence and early adulthood, a developmental stage marked by the maturation of the fronto-striatal networks. The basal ganglia, specifically the nucleus accumbens (NAcc) and the caudate nucleus (CN), are part of the fronto-striatal limbic circuit involved in reward processes underlying addictive behaviors. Abnormal NAcc and CN morphometry has been noted in alcoholics and other drug abusers, however the effects of BD on these subcortical regions have been poorly explored. Accordingly, the main goal of the present study was to address potential morphological alterations in the NAcc and CN in a sample of college binge drinkers (BDs).

**Method:**

Manual segmentation of the NAcc and the CN was performed in Magnetic Resonance Imaging (MRI) of 20 college BDs and 16 age-matched alcohol abstainers (18–23 years-old).

**Results:**

A two-way mixed ANOVA revealed no group differences in the volumetry of the CN, whereas increased NAcc volume was observed in the BD group when compared to their abstinent control peers.

**Discussion:**

These findings are in line with previous automatically segmented MRI reports highlighting abnormalities in a key region involved in drug rewarding processes in BDs.

## Introduction

Binge drinking (BD) is defined as repeated and brief episodes of high alcohol ingestion -a minimum of four drinks for women and five for men- in about 2 h interspersed with intervals of withdrawal. This phenomenon is commonly observed among adolescents and young adults mostly in the western countries (1–5). Remarkably, the average age of primary alcohol experience seems to be at 12 years old, and 13 for the first intoxication episode (6). This behavior is particularly worrying considering that early age drinking is associated with a greater risk of engaging in BD or suffers from an alcohol use disorder (AUD) in the near future (7–9). Likewise, early age drinkers seem to exhibit a higher susceptibility to the deleterious consequences of alcohol on the brain. Accordingly, several studies in both animals and humans have highlighted the major neurotoxic effects of acute intermittent alcohol consumption in the adolescent brain (10–13), especially in the late matured structures such as the prefrontal cortex (PFC) (14–17). In this sense, the youth tendency to engage in BD during adolescence has been associated with a protracted maturing course of the regions comprising this circuitry (18, 19). Apparently, the immaturity of this particular network has been proposed as underlying the reduced ability to regulate behavior, the involvement in a spectrum of hazardous situations/practices such as BD, and, ultimately, the increased susceptibility to the reinforcing properties of alcohol and other drugs (20–22).

Interestingly, frontal cortical regions and its connections to subcortical limbic areas have been described as more vulnerable to alcohol toxicity in adolescence (23–25). Specifically, the fronto-striatal limbic circuitry, involving the PFC, the anterior cingulate cortex (ACC), the amygdala, and the striatum, is well acknowledged in the field of addiction considering its key role in the development and maintenance of addictive behaviors (26, 27). Particularly, the ventral striatum -which includes the nucleus accumbens (NAcc)- has been associated with the reinforcing properties of acute alcohol consumption, whereas the CN, which is part of the dorsal striatum, seems to be more closely involved in the compulsive drug seeking behavior observed in addiction (25).

Morphometric alterations within the fronto-striatal reward pathway, namely, in the striatum nuclei, have been documented both in non-dependent and dependent substance users (e.g., 28–30). Specifically, increased NAcc volumes were observed in current alcohol and cannabis users (28, 31), while dependent alcohol or cocaine consumers, which were passing through a detoxifying process when assessed, displayed reduced NAcc volumes in relation to their age-matched healthy controls (29, 32, 33). Additionally, mixed results were observed in the CN. Whereas some studies failed to found morphometric alterations in the CN of alcoholics (29), others reported decreased volumes in this subcortical region (34).

However, despite the relevance of the striatal nuclei in addictive-like behaviors, relatively few studies have addressed the neuroanatomical substrates of these subcortical structures in young BD adults. To the best of our knowledge, to date only two studies have reported NAcc disruptions in college binge drinkers (BDs) in comparison with light drinkers. As such, using magnetic resonance imaging (MRI), Howell et al. (31) reported greater NAcc volumes in college BDs. A similar pattern of results, although gender-specific, was found by Kvamme et al. (35), namely, increased NAcc volume in BD females in comparison with non-BD females. Regarding the CN, the findings are less consistent. While some studies failed to find alterations in the BDs' CN morphometry (36), others reported decreased CN volumes in this population (30, 35).

MRI-based measurements and reproducibility are influenced by methodological factors, i.e., the image acquisition protocol, the pre/postprocessing pipeline, and the morphometric assessment, -automated v*s* manual segmentation- (37, 38). The most commonly used methods to estimate brain morphometry rely on completely automated segmentation algorithms (39) such as the voxel-based morphometry (VBM) method (40) or whole automated brain/region-of-interest (ROI) parcellation protocols with tools such as the Freesurfer or FSL (39, 41). On the other hand, manually segmented methods have long been referred as the gold standard for structural neuroanatomical measurements, generally used in small ROI segmentations/assessments, mainly for subcortical regions (e.g., 42). Clearly, both procedures have pros and cons. Specifically, the manual tracing technique is very time demanding, and requests a highly user intervention computational cost. Additionally, the ROI has to be identified and traced at each slice, limiting the application of this type of analysis to large amounts of data. Also, the precision of the segmentation might be compromised by the intra- and inter-rater variability (41, 43). Automated methods can in part counter some of these limitations by, for example, reducing the rater's subjectivity and increasing segmentation accuracy, but they largely depend on the parcellation/segmentation packages selection and preprocessing steps applied (e.g., 44). In this sense, manual tracing has some advantages in relation to the automated segmentation procedures such as more rigorous control of inter-subject variability of the brain's size and shape, superior accuracy in volume's estimation, and increased segmentation precision, especially when the ROIs' anatomical boundaries are more difficult to determine as in the subcortical regions (37, 44, 45). However, despite its potential for detecting structural alterations in addictive behaviors including alcoholism (46–48) to the best of our knowledge no study so far has manually segmented the striatal nuclei in BDs.

In this sense, we aimed to explore the structural properties of the NAcc and CN, in a group of young college BDs comparing with a group of AACs. We hypothesized an increased striatal volume in the BD group, primarily in the NAcc, when compared to the alcohol abstinent controls (AACs), in line with the previously VBM findings (31, 35).

## Method

### Participants

Initial recruitment of the potential participants to be enrolled in the present study was carried out throughout an online survey and it was based on the current alcohol and other substances consumption, and age range (18–23 years old). This first selection was based on the BD definition established by the National Institute on Alcohol abuse and Alcoholism (4) -a minimum of five standard drinks (four for women) in a short period of time (2 h)- at least once per month, with a minimum duration of 10 months for the BD group. As for the control group, the criterion defined was not consuming alcohol either now or in the past.

Subsequently, a clinical interview and a behavioral assessment were conducted to assess whether the preselected participants were eligible to participate in the study. The assessment protocol included the Portuguese version of the Alcohol Use Disorder Identification Test (AUDIT) (49, 50), the Alcohol Use Questionnaire (AUQ) (51), the Semi-Structured Assessment for the Genetics of Alcoholism (SSAGA), the Individual Assessment Module (IAM), the Family History Assessment Module (FHAM) (52), the Portuguese version of the Symptom Checklist-90 revised questionnaire (SCL-90-R) (53, 54), and the Edinburg Handedness Inventory (55).

Participants with history of traumatic brain injury or neurological disorder, personal and/or family history of DSM-IV-R axis I disorder or alcoholism in first-degree relatives, scores > 90 for the global severity index (GSI) or at least 2 symptomatic dimensions for the SCL-90-R, scores ≥ 20 in the AUDIT or AUD based on the DSM-IV-R criteria, regular or occasional use of other drugs including prescribed psychoactive substances (except occasional cannabis use), uncorrected sensory deficits or left handedness were excluded from the study. Tobacco consumption was not defined as an exclusion criterion since a high correlation between alcohol and tobacco consumption in adolescents and university students has been consistently reported in the literature (56–60).

Finally, 36 college students were included in the study. Twenty individuals were assigned to the BD group and 16 alcohol-abstinent individuals composed the AAC group. **Table 1** displays the demographics and alcohol-related characteristics for both groups.

**Table 1:** Demographic and behavioral data for binge drinkers and alcohol abstinent controls.

	BD N = 20 Mean (SD)	AAC N = 16 Mean (SD)	t (34)	
% Male	50%	37,5%	z = -0.740
% Female	50%	62,5%	
% Caucasian	100	100	
Age	20.45 (1.60)	21.00 (1.71)	.99
Age of BD onset	17.45 (1.08)	—	
AUDIT (total score)	11.20 ± 3.25	.62 ± 1.20	-13.43***
Number of times of BD per month	3.57 ± 1.87	0	-8.54***
Number of months with BD pattern	35.90 ± 14.03	0	-11.44***
Grams of alcohol consumed per week	151 ± 44.27	0	-14.78***
Speed of drinking (gr/h during BD episodes)	34.50 ± 8.26	0	-18.69***
Percentage of times getting drunk when drinking	43.25 ± 20.41	0	-9.48***
Tobacco Smokers	7	0	
Occasional users of Cannabis	2	0	

AUDIT, Alcohol Use Disorders Identification Test; BD, binge drinking; AAC, alcohol-abstinent control; SD, standard deviation.

All p-values reported are for 2-tailed independent samples t-tests, except for the variable gender for which a Mann-Whitney test was calculated.

***P < 0.001.

Previous to the MRI scanning, individuals were asked to not engage in BD episodes for the three preceding days, not consuming alcohol for at least 12 h before the MRI assessment, and avoid caffeinated beverages ingestion and smoking for at least 3 h in advance.

The research protocol was designed considering the ethical principles for medical research involving human subjects of the World Medical Association (WMA) present in the Declaration of Helsinki (61) and approved by the Portuguese Bioethics Committee of the University of Minho. Participants were informed about the research procedure, gave written informed consent and received a financial stipend.

### Magnetic Resonance Image Acquisition

Before the MRI acquisition all the participants were screened for possible contraindications that could interact with the scanner magnetic field (e.g. earrings, hair hooks, keys, etc.) and affect their safety. Participants were then familiarized with the scanner and instructed on the procedure. At the time of the acquisition, supported by both the hospital technician and the investigator, the participants were comfortably placed in the scanner table with head and foot support, and noise-reducing headphones. The study instructions were again revised and the participant was given a call button to activate an alert in case they needed help and wanted to finish the session.

Sagittal high-resolution 3D T1 weighted anatomical images were acquired in a Siemens Magneton TrioTim 3T MRI scanner (Siemens Medical Solutions, Erlangen, Germany) equipped with a 32-channel receive-only head coil, and software Version Syngo MR D12 applying a magnetization prepared rapid acquisition gradient echo (MPRAGE) ascending interleaved sequence and parameters: repetition time (TR) = 2,700 ms, echo time (TE) = 2.33 ms, inversion time (TI) = 1,000 ms, delay time (TD) = 1,600 ms, flip angle (FA) = 7°, 192 slices with 0.8 mm thickness, slab thickness = 153.6 mm, slice gap = 0 mm, in-plane resolution = 1x1 mm^2^, matrix size = 320x310 and 256 mm field of view (FoV). The total acquisition time was 6.49 min.

### Image Preprocessing

After the acquisition, all MRI scans were visually controlled to discard for critical head motion or brain lesions. After this verification, the 3D-Slicer Version 4.10 image-editing tool (http://www.slicer.org) was used to perform image segmentation. Manual tracing of the ROIs was performed on the T1 MRI native space. Each ROI was computed as the sum of all the voxels included in the specific ROIs under assessment. Total intracranial volume (TIV) was calculated as the sum of gray matter, white matter and CSF volumes using the 3D-Slicer Version 4.10 EMSegmenter tool.

### Region of Interest Definition

**Figure 1:**
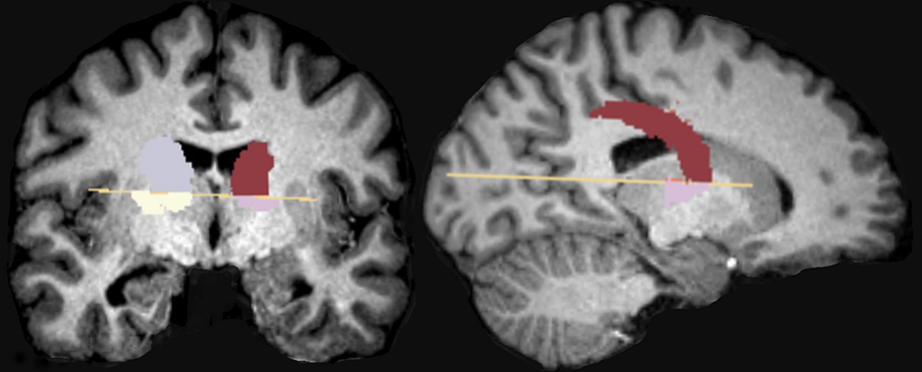
Represents the NAcc and CN 2D reconstruction.

The region of interest (ROI) included the NAcc and CN. The manual segmentation protocol was the one proposed by Levitt et al. (62). Accordingly, a large polygon was draw on the axial view of the anterior commissure–posterior commissure (AC–PC) plane, used as a landmark to trace the NAcc. To delineate the NAcc, we drew two intersecting lines, the first one skimming the superior surfaces of the caudate and putamen, and the second drawn along the plane formed by the internal capsule–putamen border and intersecting the first (forming an “X”). A vertical line from the intersection of this “X” was drawn to the ventrolateral border of the putamen, marking Point A. To define the dorsal boundary of the NAcc, the midpoint between the medial edge of the caudate and lateral edge of the putamen at the level of AC–PC plane was marked (Point B). The NAcc was drawn anteriorly until it was no longer present at or below the level of the AC–PC plane. The CN were measured bilaterally, using three orthogonal planes, in all slices in which they appeared. **Figure 1** shows the 2D reconstruction of both structures — see Coutinho et al. (63) and Levitt et al. (62) for more details regarding the anatomical landmarks. Manual segmentation was performed with 3D-Slicer (http://www.slicer.org).

### Data Analysis and Procedure

One rater segmented the NAcc and CN of the total sample (36 participants). Other researcher with experience in manual segmentation traced the NAcc and CN of 11 randomly assigned cases, corresponding to 30.5% of the sample. Both raters were blinded to the participant's diagnosis.

The NAcc and CN were manually segmented in 18 slices following the protocol referred above. The volumes of each ROI were computed as the sum of all the voxels included in the specific ROIs under assessment. The NAcc and CN volumes, plus TIV, total gray and white matter volumes were then extracted and exported to a SPSS database and statistical analyses were conducted for the NAcc and CN volumes (left and right hemispheres), plus TIV, total gray and white matter volumes using SPSS package Version 20.0.

### Statistical Analysis

The Intraclass correlation reliability between the two raters was assessed by Cronbach's alpha, and Student's t-tests were used to compare the TIV, total gray and total white matter volumes between groups. Statistical significance was defined as p ≤ 0.05.

In order to correct for head size variation, the relative volumes were calculated for each ROI -absolute volumes divided by TIV and multiplied by 100.

 A two-way mixed ANOVA was calculated for each ROI (NAcc and CN) with group (BD vs AAC) and gender (male vs female) as the between-subjects factors, hemisphere (left vs right) as the within-subjects factor, and age as covariate. When appropriate, degrees of freedom were corrected with the Greenhouse-Geisser procedure. False discovery rate (FDR) corrections were applied to the main and interactions effects, and post-hoc paired comparisons were performed with the Bonferroni adjustment for multiple comparisons (alpha level ≤ 0.05).

## Results

### Control Measures

The Intraclass correlation reliability for the right and left CN and the right and left NAcc volumes by the two raters was good or excellent: .91 and .87, and .93 and .94, respectively.

Mean values for the TIV, total gray and total white matter volumes, absolute and relative volumes of the NAcc and CN for each group are presented in **Table 2**. Control analyses revealed no between group differences in the TIV [t (34) = 1.61, p = 0.12], total gray [t (34) = 1.38, p = 0.18] and white [t (34) = 1.02, p = 0.31] matter volumes.

**Table 2:** Means and SDs for the TIV, total GM and WM volumes, absolute and relative NAcc and CN volumes for each group.

Volumes (ml) Mean (SD)											
GM	WM	TIV		NAcc Abs (ml)		NAcc Rel (ml)		CN Abs (ml)		CN Rel (ml)	
				Left	Right	Left	Right	Left	Right	Left	Right
BDs	897.23 (86.61)	507.57 (65.14)	1581.48 (158,15)	1.25 (0.15)	1.16 (0.19)	0.079 (0.008)	0.073 (0.012)	4.97 (0.58)	4.64 (0.64)	0.31 (0.02)	0.29 (0.03)
AACs	857.19 (85.94)	484.21 (71.58)	1496.19 (157.69)	0.93 (0.34)	0.80 (0.23)	0.063 (0.025)	0.054 (0.016)	4.65 (0.63)	4.33 (0.62)	0.31 (0.03)	0.29 (0.3)

AACs, alcohol abstinent controls; BDs, Binge Drinkers; CN Abs, Caudate Nucleus Absolute; CN Rel, Caudate Nucleus Relative; GM, Grey Matter; NAcc Abs, Nucleus Accumbens Absolute; NAcc Rel, Nucleus Accumbens Relative; TIV, Total Intracranial Volume; WM, White Matter.

### NAcc Volume

**Figure 2:**
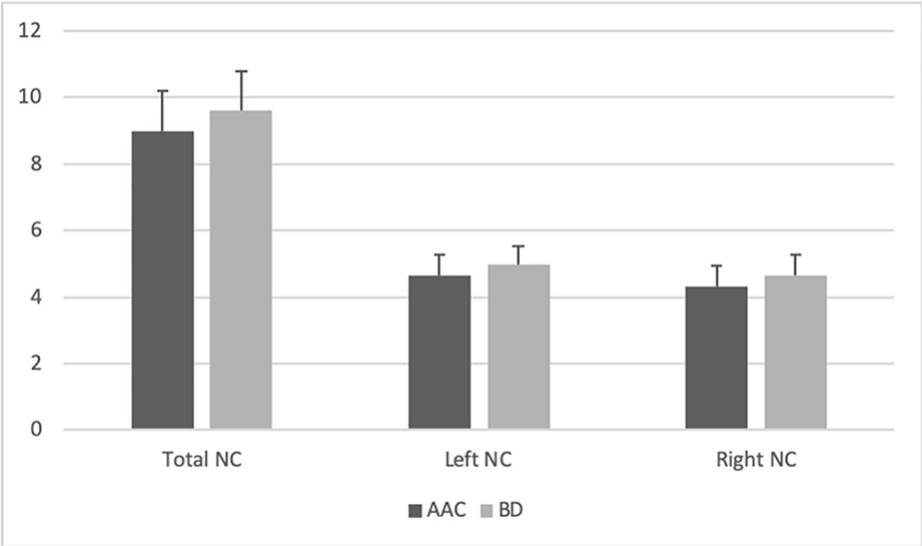
Illustrates the CN mean volumes (ml) in the BD and AAC groups. Error bars represent standard deviations.

A main effect of group revealed increased absolute NAcc volumes in the BDs [F (1,31) = 22.82, p_FDR_ < 0.001, η_p_^2^ = 0.42] when compared to their AAC peers (see **Figure 2**). Additionally, no significant Group x Gender [F (1,31) = 2.68, p_FDR_ = 0.30] or Group x Hemisphere [F (1,31) = 0.07, p_FDR_ = 0.93] interaction effects were observed. This pattern was also observed for relative NAcc volumes (to TIV), with a group effect [F (1,31) = 16.16, p_FDR_ < 0.01, η_p_^2^ = 0.34] and increased relative NAcc volumes in BDs, with no Group x Gender [F (1,31) = 1.01, p_FDR_ = 0.45] or Group x Hemisphere [F (1,31) = 0.01, p_FDR_ = 0.93] interaction effects being observed.

In order to control the potential effect of tobacco and occasional cannabis consumption in the results, the same analyses were conducted but excluding those BD participants who consumed tobacco and occasional cannabis. Similar significant group differences in the NAcc were found between BDs and AACs, both in the relative [F (1,23) = 9.43, p_FDR_ = 0.05, η_p_^2^ = 0.29] and absolute volumes [F (1,23) = 14.84, p_FDR_ = 0.01, η_p_^2^ = 0.39]. Additionally, no group differences were observed when comparing BDs without any other consumption and BDs with tobacco and occasionally cannabis consumption.

### CN Volume

**Figure 3:**
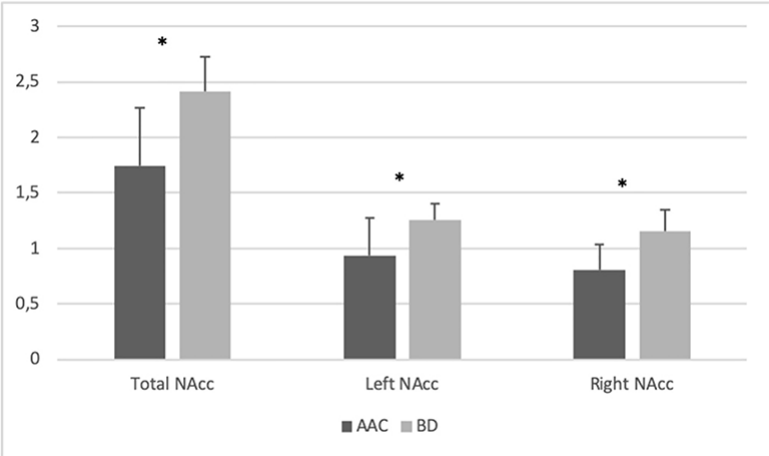
Illustrates the NAcc mean volumes (ml) in the BD and AAC groups. Error bars represent standard deviations. *P ≤ 0.001

No significant between-group differences were found in the absolute or relative CN volumes [absolute: [F (1, 31) = 1.13, p_FDR_ = 0.74]; relative [F (1, 31) = 0.09, p_FDR_ = 1.00] (see **Figure 3**). Similarly, no Group x Gender [absolute: F (1,31) = 0.34, p_FDR_ = 0.84; relative [F (1,31) = 0.05, p_FDR_ = 1.00] or Group x Hemisphere [absolute: F (1,31) = 0.01, p_FDR_ = 0.97; relative [F (1,31) = 0.02, p_FDR_ = 1.00] interaction effects were noted.

## Discussion

This study assessed the NAcc and CN volumes in a group of college BDs in comparison with age-matched alcohol-abstainers using a manually segmented MRI protocol. Our results showed that BDs displayed greater bilateral gray matter (absolute and relative) volumes in the NAcc, whereas no between-group differences were observed in the CN, when compared to the AAC group.

The present findings are consistent with recent evidence showing increased NAcc volumes in college-aged BDs (31, 35). Specifically, Howell et al. (31) observed a greater NAcc volume in the BD group, relative to healthy controls, and suggested that this could represent a neuroanatomical immaturity associated with the binge alcohol use. Kvamme et al. (35) also observed an (gender-specific) increase in the NAcc volume of female BDs, a result that was again interpreted as a potential alcohol-induced deleterious effect on the neuromaturation trajectories of young BDs.

Developmental research has shown that subcortical gray matter volumes seem to peak during early adolescence, typically following an inverted U-shaped developmental trajectory (64). This natural decline in subcortical volume throughout adolescence – mostly driven by synaptic pruning (65) – has been reported as being disrupted by substance-induced toxicity, such as alcohol or cannabis (28, 31, 35). Likewise, maladaptive structural plasticity of NAcc neurons has also been proposed as a significant feature of excessive alcohol use, observed both in animal and human studies (66). In particular, exposure to intermittent alcohol administration in animal models has shown to increase dendritic branching and spine density in the mice NAcc (67, 68). Additionally, a recent study found higher orientation dispersion index (ODI) -a measure that captures the architecture of dendritic processes- in the ventral striatum of young adult BDs when compared to healthy volunteers (69). A positive association between the ODI and binge score (but not with the AUDIT) was further observed in BDs, suggesting that dendritic modifications –increased dendritic complexity – might be better explained by a binge pattern of alcohol consumption rather than by the severity of alcohol use (69).

Collectively, these studies open the discussion to two hypotheses. One suggests that BD at young ages may interfere with the neuromaturational processes (e.g., dendritic arborization, synaptic pruning) taking place in subcortical regions such as the NAcc. This interpretation of a neuromaturational delay has also been suggested from other studies showing increased gray matter volume in frontal regions in female (35, 70) or in both female and male BDs (71, 72). The other hypothesis suggests that pre-existing neuroanatomical differences may constitute a risk factor for BD, as documented by recent longitudinal studies (73, 74). Specifically, adolescents (females, but not males) with greater NAcc volume at baseline were more likely to binge drink 2 years later (73), thus suggesting that delayed structural maturation of the NAcc may predispose towards excessive alcohol use in adolescence. Likewise, another study conducted by the same research group reported that adolescents with lower premorbid fractional anisotropy – an index of white-matter microstructure complexity – in pathways connecting the NAcc to frontal regions began BD sooner, suggesting again that a delayed maturation of accumbofrontal connections may represent a premorbid risk factor for earlier initiation of heavy alcohol use (74). Taken together, these mixed results come to confirm the need for new research, particularly longitudinal studies, in order to better disentangle the relationship between prefrontal/subcortical volume alterations and previous/subsequent alcohol use.

Regarding the CN, no significant between-group differences were observed in our study. Previous studies have provided little consistency when examining the potential abnormalities of this region in young binge or heavy drinkers. Specifically, in line with our findings, the co-twin study conducted by Wilson et al. (36) also failed to find changes in the CN morphometry of adolescent BDs, while others (30, 35) documented decreased CN volumes in this population. Accordingly, additional research is necessary to clarify the mixed CN findings reported in BD considering the involvement of this structure in the compulsive drug seeking behavior observed in addiction (75).

 Overall, our findings add support to the assumption that morphological modifications in brain regions engaged in the addiction cycle (25) are present in BDs. Different roles of the dorsal and the ventral striatum have been proposed in the pathways that mediate the three stages of the addiction cycle. Specifically, the ventral striatum seems to be involved in the binge/intoxication phase characterized by the acute administration/use of drugs, which elicits a rewarding response (76), whereas the dorsal striatum appears to be recruited during the stimulus-response habit learning contributing to the development of compulsive drug-seeking behavior. Therefore, the instrumental function of each of these structures might be mediating the mixed results found in the studies as well as our observations. In addition, it has been reported that not only the NAcc, but also prefrontal regions of the fronto-striatal reward circuitry have shown to be structurally and functionally impaired in college BDs (e.g., 35, 73, 77–82), suggesting that alterations of the fronto-striatal reward network might be prompting or contributing to initiating/maintaining the binge pattern of alcohol consumption among youth.

Finally, this study displays some limitations that deserve consideration when interpreting the results. Firstly, the cross-sectional nature of the present study precludes us to determine whether differences in the NAcc volume precede alcohol use or are a consequence of BD. Secondly, the limited sample size of our study could also be an important factor that may undermine the reliability of the findings. Finally, the participants enrolled in our study are all college students, what might also influence the generalizability of our results.

In summary, the present study used a manually segmented MRI analysis to evaluate potential differences in the NAcc and the CN volumetry between college alcohol abstainers and age-matched BDs. Results revealed that the NAcc volume was significantly increased in the BD group in comparison with the AAC group. These findings seem to be in line with prior automatically segmented MRI studies carried out in young BDs and are suggestive of delayed structural maturation of the NAcc. Further research will be needed to determine whether these anomalies precede (and therefore constitute a risk factor for) alcohol use or instead are a consequence of BD.

## Data Availability Statement

The datasets generated for this study are available on request to the corresponding author.

## Ethics Statement

The studies involving human participants were reviewed and approved by Portuguese Bioethics Committee of the University of Minho. The patients/participants provided their written informed consent to participate in this study.

## Author Contributions

SS collaborated in the statistical analysis, interpreted the results, wrote the manuscript, carried out subject's recruitment and assessment, and participated in data acquisition and processing. AS coordinated data acquisition, collaborated in the data processing, statistical analysis and in manuscript writing. EL-C collaborated in interpretation of the results and manuscript writing. CB performed the manual segmentation protocol. ÓG collaborated in manuscript writing and AC designed the study, coordinated subject's recruitment, assessment and data acquisition, carried out the statistical analysis, and collaborated in manuscript writing. All authors read and approved the final manuscript.

## Funding

This study was conducted at the Psychology Research Centre (PSI/01662), School of Psychology, University of Minho, and supported by the Portuguese Foundation for Science and Technology and the Portuguese Ministry of Science, Technology and Higher Education (UID/PSI/01662/2019), through the national funds (PIDDAC). This study was also supported by the project POCI-01-0145-FEDER-028672, funded by the Portuguese Foundation for Science and Technology (FCT) and the European Regional Development Fund (FEDER). SS was supported by the SFRH/BD/88628/2012, Doctoral Fellowship of the Portuguese Foundation for Science and Technology, co-financed by POPH/FSE through QREN. EL-C and AC were supported by the FCT and the Portuguese Ministry of Science, Technology and Higher Education, through the national funds, within the scope of the Transitory Disposition of the Decrete No. 57/2016, of 29th of August, amended by Law No. 57/2017 of 19 July.

## Conflict of Interest

The authors declare that the research was conducted in the absence of any commercial or financial relationships that could be construed as a potential conflict of interest.

## Abbreviations

AC–PC, Anterior commissure–posterior commissure; AUD, Alcohol Use Disorders; AUDIT, Alcohol Use Disorder Identification Test; AUDs, individuals with alcohol use disorders; BD, Binge drinking; BDs, Binge Drinkers; CN, Nucleus Caudate; MRI, Magnetic Resonance Imaging; NAcc, Nucleus Accumbens; ODI, Orientation dispersion index; ROI, Region-of-interest; ROIs, Regions-of-interest; TIV, Total intracranial volumes; VBM, Voxel-based Morphometry.
